# Exploring the Interaction between E484K and N501Y Substitutions of SARS-CoV-2 in Shaping the Transmission Advantage of COVID-19 in Brazil: A Modeling Study

**DOI:** 10.4269/ajtmh.21-0412

**Published:** 2021-09-27

**Authors:** Shi Zhao, Jinjun Ran, Lefei Han

**Affiliations:** ^1^ JC School of Public Health and Primary Care, Chinese University of Hong Kong, Hong Kong, China;; ^2^ CUHK Shenzhen Research Institute, Shenzhen, China;; ^3^ School of Public Health, Shanghai Jiao Tong University School of Medicine, Shanghai, China;; ^4^ School of Global Health, Chinese Center for Tropical Diseases Research, Shanghai Jiao Tong University School of Medicine, Shanghai, China

## Abstract

The COVID-19 pandemic poses serious threats to global health, and the emerging mutation in SARS-CoV-2 genomes is one of the major challenges of disease control. Considering the growth of epidemic curve and the circulating SARS-CoV-2 variants in Brazil, the role of locally prevalent E484K and N501Y substitutions in contributing to the epidemiological outcomes is of public health interest for investigation. We developed a likelihood-based statistical framework to reconstruct reproduction numbers, estimate transmission advantage associated with different SARS-CoV-2 variants regarding the marking (identifying) 484K and 501Y substitutions (including Alpha, Zeta, and Gamma variants) in Brazil, and explored the interactive effects of genetic activities on transmission advantage marked by these two mutations. We found a significant transmission advantage associated with the 484K/501Y variants (including P.1 or Gamma variants), which increased the infectivity significantly by 23%. In contrast and by comparison to Gamma variants, E484K or N501Y (including Alpha or Zeta variants) substitution alone appeared less likely to secure a concrete transmission advantage in Brazil. Our finding indicates that the combined impact of genetic activities on transmission advantage marked by 484K/501Y outperforms their independent contributions in Brazil, which implies an interactive effect in shaping the increase in the infectivity of COVID-19. Future studies are needed to investigate the mechanisms of how E484K and N501Y mutations and the complex genetic mutation activities marked by them in SARS-CoV-2 affect the transmissibility of COVID-19.

## INTRODUCTION

COVID-19, the etiological agent of which is severe acute respiratory syndrome coronavirus 2 (SARS-CoV-2),^1^ posed a serious threat to global health and swept the world in 2020; the pandemic is still ongoing.^2^^,^^3^ As of March 31, 2021, more than 127 million COVID-19 cases had been confirmed worldwide, with more than 2.7 million associated deaths.

The control of COVID-19 requires knowledge of the driving factors that may affect the transmission process^4^^,^^5^; virus mutation is one of the major challenges.^6^^,^^7^ Around September 2020, genetic variants carrying the N501Y substitution on the spike (S) protein of SARS-CoV-2 were first detected in the United Kingdom^8^ and, then spread globally and trended to reach fixation rapidly in many places (e.g., South Africa,^9^ Brazil,^10^ the United States,^11^ and the United Kingdom).^12^^,^^13^ In Brazil, the 501Y variants, as well as other amino acid changes, were clustered into the B.1.1.28.1 lineage by COVID-19 Genomics Consortium UK, which is also known as the variant of concern (VoC) 202101/02.^14^ The B.1.1.28.1 lineage is a descendant of the B.1.1.28 lineage that has another similar descendant lineage, B.1.1.28.2, carries the E484K substitution but not N501Y substitution.^15^^,^^16^ The mutation E484K was first identified in South Africa and became prevalent in many places, including the United Kingdome and Brazil.^17^ These emerging variants may affect the epidemiological characteristics of COVID-19^18^^,^^19^ and the protective effects of vaccines.^20^^–^^23^ Considering the growing patterns of the epidemic curve in Brazil, the possible contributions of both E484K and N501Y substitutions are of public health interest for investigation.

The rapid spread of 501Y and 484K variants indicates a possible transmission advantage over their preceding variants.^24^ On one hand, recent analyses reported evidence that the N501Y substitution was associated with an increase in infectivity of COVID-19,^12^^,^^13^^,^^25^^–^^27^ which appears similar to the situation of D614G substitution reported previously.^28^^–^^31^ On the other hand, the relationship between E484K substitution and COVID-19 transmissibility appears inconclusive.^32^^,^^33^ The survival or functional profile of pathogen could be altered through genetic mutation and, as a consequence, change its infectivity.^34^ Referring to the previous studies on seasonal influenza viruses,^35^ a few key amino acid substitutions may lead to changes in antigenic features and epidemiological outcomes,^36^^,^^37^ and the interaction among them may become more complicated. As an example, the R384G substitution in the nucleoprotein (NP) of H3N2 virus enhances the ability of in-host immune escape,^38^ which increases transmissibility,^35^ but this substitution appears detrimental. In contrast, the commutations including E375G and M239V in NP could compensate and restore the viral fitness or functionality of H3N2 virus,^39^^,^^40^ just as the mutated strains rapidly reached fixation in 1993–1994 influenza season. For the COVID-19 epidemics in Brazil, how the mutation activities marked by E484K and N501Y substitutions, as well as the possible interactive effect between them, might shape the transmission advantage remains unassessed.

Exploring the role of mutation activities in determining disease transmissibility is of importance to understand how the evolutionary process at molecular scale may shape the epidemiological outcomes at population scale.^28^^,^^31^^,^^41^^,^^42^ In this study, we adopt a statistical framework to infer the real-time transmissibility associated with different SARS-CoV-2 variants with respect to E484K and N501Y substitutions in Brazil. We explore the interactive effects between E484K and N501Y in shaping the transmission advantage of COVID-19.

## METHODS

### SARS-CoV-2 sequencing data and COVID-19 surveillance data.

The SARS-CoV-2 strains were obtained via the global initiative on sharing all influenza data (GISAID) with collection dates ranging from January 1, 2020 to January 31, 2021, in the Brazil.^43^ A total of 4,210 complete human SARS-CoV-2 strains were retrieved. We excluded sequences with more than 5% ambiguous amino acids during the alignment, and a total of 4,052 sequences were included for further analysis. Multiple sequence alignment was performed using MAFFT version 7,^44^ and the “Wuhan-Hu-1” (GISAID: EPI_ISL_402125 or GenBank: NC_045512.2) SARS-CoV-2 genome is considered as the reference sequence.

**Figure 1.**
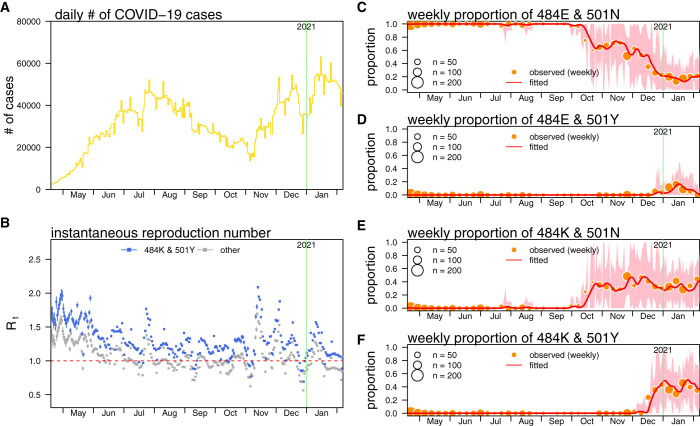
(**A**) The daily number of COVID-19 cases in Brazil, (**B**) the reconstructed reproduction number (*R_t_*), (**C**) proportions of the 484E/501N variants, (**D**) 484E/501Y variants (including B.1.1.7 or Alpha variants), (**E**) 484K/501N variants (including P.2 or Zeta variants), and (**F**) 484K/501Y variants (including P.1 or Gamma variants). Panel B shows the estimated *R_t_*s of 484K/501Y variants (in blue) and the other three types of variants (in gray). The dots are the estimates, and bars are the 95% confidence intervals (CIs). In panels C through F, the dots are the observations, the curve indicates the mean fitting results, and the shading area indicates the 95% CI. This figure appears in color at www.ajtmh.org.

The surveillance data of COVID-19 cases in the Brazil were collected via the WHO COVID-19 surveillance platform.^45^ To avoid the under-ascertainment due to reporting delay, we drop the observations since February 2021. As such, the surveillance data of COVID-19 cases from January 1, 2020 to January 31, 2021, are included in the analysis, which match the period of SARS-CoV-2 sequencing data. To adjust for the weekly cycle in the COVID-19 case time series, the 7-day moving average is adopted for further analysis. The COVID-19 cases time series are shown in Figure 1A.

### Statistical parameterization.

#### Variant-specific reproduction number.

The time-varying reproduction number is commonly adopted to quantify the instantaneous transmissibility of infectious disease in an epidemic. Using the estimation framework in Cori et al.,^46^ the epidemic growth is modeled as a branching process. Thus, the reproduction number at time *t*, *R*(*t*), is expressed as the ratio of *C*(*t*) over $\int_{0}^{\infty }w\left(\tau \right)C\left(t-\tau \right)\text{d}\tau$, which is commonly known as the renewable equation.^47^^,^^48^ Here, the *C*(*t*) is the observed new incidences of COVID-19 at time *t*. The function *w*(·) is the distribution of the generation time (GT) of the disease, that is, COVID-19. The GT is defined as the time interval between the time of exposure, that is, being infected, of a primary case and that of his associated secondary case in the consecutive transmission generation.^49^ Thus, the distribution *w*(·) is predefined in our model, which is commonly estimated from contract tracing surveillance data.^50^^–^^53^ To set up the analysis for COVID-19, we consider *w* as a Gamma distribution having mean (±SD) values of 5.3 (±2.1) days by averaging the GT estimates from the existing literatures.^50^^–^^57^ Slight variation in the settings of the GT will not affect our main findings.

To incorporate the information of SARS-CoV-2 variants, we denote the proportion (or prevalence) of the *j*-th variant of concern (VoC) at calendar time *t* by ρ*_j_*(*t*), which is time-varying. Straightforwardly, $\sum_{j}^{}\rho_{j}(t)=1$ for all *t*. We denote the variant-specific reproduction number for the *j*-th VoC at time *t* by *R_j_*(*t*), and we have $R_{j}(t)=\frac{\rho_{j}\left(t\right)C(t)}{\int_{0}^{\infty }w\left(\tau \right)\rho_{j}\left(t-\tau \right)C\left(t-\tau \right)\text{d}\tau }$. We consider the expected new incidences infected by *j*-th VoC at time *t*, denoted by **E**[*C_j_*(*t*)], which can be modeled in Eq. (1).

$$E\left[C_{j}(t)\right]=R_{j}(t)\int_{0}^{\infty }w\left(\tau \right)\rho_{j}\left(t-\tau \right)C\left(t-\tau \right)\text{d}\tau .$$ (1)

Equation (1) will be used to formulate the likelihood function in the remaining sections. Note that the index *j* is merely used for labeling instead of ranking.

#### Transmission advantages and their interactive effects.

For convenience, we label the original variant as the (*j* =) 0-th VoC, that is, the 484E/501N variant, and thus its associated variant-specific reproduction number is *R_j_*_= 0_. Similarly, we label the 484E/501Y, 484K/501N, and 484K/501Y variants as the *j* = 1, 2, and 3 VoC, respectively.

Following previous work,^28^^,^^58^ the transmission advantage of the mutated variant against the original type is defined as the ratio (denoted by η) of the strain-specified reproduction numbers. Considering the transmissibility of the original 484E/501N variant (*R_j_*_ = 0_) as the reference level, the transmission advantage of the *j*-th VoC is $\eta_{j}=\frac{R_{j}}{R_{j=0}}$. Thus, the reproduction number of cases infected by the *j*-th VoC is *R_j_* = η*_j_*·*R_j_*_=0_, and η*_j_*_= 0_ = 1 by definition. If η*_j_* > 1, the *j*-th variant may be more infectious than the original genetic variant, and *vice versa*. In addition, the overall reproduction number is $R_{j=0}(t)\sum_{j}^{}\left[\eta_{j}\rho_{j}\left(t\right)\right]$.

We consider η*_j_* as a constant, which reflects an intrinsic nature of the *j*-th SARS-CoV-2 variant, and thus η*_j_* is invariant with time. Hence, we have *R_j_*(*t*) = η*_j_*·*R_j_*_=0_(*t*) for all time *t*. Then, we calculate the expected number of COVID-19 cases, **E**[*C*(*t*)], at time *t* in Eq. (2).

$$E\left[C(t)\right]=R_{j=0}(t)\sum_{j}\left[\eta_{j}\int_{0}^{\infty }w\left(\tau \right)\rho_{j}\left(t-\tau \right)C\left(t-\tau \right)\text{d}\tau \right].$$ (2)

As such, for the observed sequencing data, the expected chance (or probability) that a randomly selected strain at the *t*-th day is *j*-th VoC, **E**[ρ*_j_*(*t*)], is given in Eq. (3).

$$E\left[\rho_{j}(t)\right]=\frac{E\left[C_{j}(t)\right]}{E\left[C(t)\right]}=\frac{\eta_{j}\int_{0}^{\infty }w\left(\tau \right)\rho_{j}\left(t-\tau \right)C\left(t-\tau \right)\text{d}\tau }{\sum_{j}\left[\eta_{j}\int_{0}^{\infty }w\left(\tau \right)\rho_{j}\left(t-\tau \right)C\left(t-\tau \right)\text{d}\tau \right]}.$$ (3)

Straightforwardly, $\sum_{j}E\left[\rho_{j}(t)\right]=1$ for all *t*.

For the E484K and N501Y substitutions, the 484E/501Y (*j* = 1, i.e., including the B.1.1.7 or Alpha variants) and 484K/501N (*j* = 2, i.e., including the P.2 or Zeta variants) are two variants with merely one substitution, whereas the 484K/501Y variant (*j* = 3, i.e., including the P.1 or Gamma variants) has both substitutions. To explore the interactive effects on the variant-specific reproduction number, we compare η*_j_*_=3_ and the product of (η*_j_*_=1_·η*_j_*_=2_). If η*_j_*_=3_ is larger than (η*_j_*_=1_·η*_j_*_=2_), the E484K and N501Y substitutions may enhance the transmissibility than their separated partial effects, and vice versa.

### Likelihood-based inference.

According to Eq. (2), we construct the likelihood function $L_{t}^{\left(\text{c}\right)}$ of the daily number of cases using a Poisson-distributed framework with observation at *C_t_* and rate parameter at **E**[*C_t_*] as in Eq. (4).

$$L_{t}^{\left(\text{c}\right)}\left(C_{t}|E\left[C_{t}\right]\right)=\frac{E{\left[C_{t}\right]}^{C_{t}}\cdot e^{-E\left[C_{t}\right]}}{C_{t}!}.$$ (4)

Here, the *C_t_* is the observed number of COVID-19 cases on day *t* and is the discretized *C*(*t*), which means $C_{t}=\int_{\text{day }\;t}C\left(x\right)\text{d}x$. The value of *C_t_* can be obtained from the number of COVID-19 cases time series as shown in Figure 1A. Note that the superscript ^(c)^ merely indicates that the likelihood function is for the number of cases, which does not indicate the power.

For the observed sequencing data, we denote the numbers of *j* = 0, 1, 2, and 3 variants by *m_j_*_=0,_*_t_*, *m_j_*_=1,_*_t_*, *m_j_*_=2,_*_t_*, and *m_j_*_=3,_*_t_*, respectively, for the day *t*. Thus, we model the sampling process of genetic variants using a generalized Bernoulli distribution, that is, categorical distribution, with probabilities at **E**[ρ*_j_*(*t*)]s in Eq. (3). The likelihood function $L_{t}^{\left(\text{s}\right)}$ is constructed in Eq. (5).

$$L_{t}^{\left(s\right)}\left(m_{j=0,t},\ldots ,m_{j=3,t}|E\left[\rho_{j=0,t}\right],\ldots ,E\left[\rho_{j=3,t}\right]\right)=\prod_{j}E{\left[\rho_{j,t}\right]}^{m_{j,t}}.$$ (5)

Here, the **E**[ρ*_j_*_,_*_t_*] is the expectation of variant prevalence for day *t*. Note that the superscript ^(s)^ merely indicates that the likelihood function is for genetic variants, which does not indicate the power.

With Eqs. (4) and (5), we reconstruct the *R_j_*_=0,_*_t_* time series, denoted by $\left\{R_{j=0,t}\right\}$, and estimate η*_j_*_=1_, η*_j_*_=2_, and η*_j_*_=3_ using the overall log-likelihood function *ℓ* defined in Eq. (6).

$$\ell \left(\left\{R_{j=0,t}\right\},\eta_{j=1},\eta_{j=2},\eta_{j=3}|\left\{C_{t}\right\},\left\{m_{j=0,t}\right\},\ldots ,\left\{m_{j=3,t}\right\}\right)=\sum_{t}\log\left[L_{t}^{\left(c\right)}\times L_{t}^{\left(s\right)}\right].$$ (6)

We calculate the maximum likelihood estimates (MLE) of parameters to determine transmission advantage of 484E/501Y (η*_j_*_=1_), 484K/501N (η*_j_*_=2_), and 484K/501Y (η*_j_*_=3_) variants by using the likelihood framework defined in Eq. (6). The 95% confidence intervals (95% CI) are calculated using the profile likelihood estimation framework with a χ^2^ quantile as the cutoff,^59^^,^^60^ which has also been adopted in previous work.^61^^–^^67^

### Fitting schemes and their selection.

We explore the interactive effects between E484K and N501Y substitutions of SARS-CoV-2 in shaping the transmission advantage in Brazil. We consider the following eight fitting scenarios with respect to η*_j_* and investigate the role of E484K and N501Y substitutions contributing to the transmissibility of COVID-19:

• all of η*_j_*_=1_, η*_j_*_=2_, and η*_j_*_=3_ are assumed at 1:
  scenario (#1): η*_j_*_=1_ = η*_j_*_=2_ = η*_j_*_=3_ = 1;
• two of η*_j_*_=1_, η*_j_*_=2_, or η*_j_*_=3_ are assumed at 1, and the remaining one is freely estimated:
  scenario (#2): η*_j_*_=1_ = η*_j_*_=2_ = 1,
  scenario (#3): η*_j_*_=1_ = η*_j_*_=3_ = 1, and
  scenario (#4): η*_j_*_=2_ = η*_j_*_=3_ = 1;
• one of η*_j_*_=1_, η*_j_*_=2_, or η*_j_*_=3_ is assumed at 1, and the remaining two are freely estimated:
  scenario (#5): η*_j_*_=1_ = 1,
  scenario (#6): η*_j_*_=2_ = 1, and
  scenario (#7): η*_j_*_=3_ = 1;
• none of η*_j_*_=1_, η*_j_*_=2_, or η*_j_*_=3_ is assumed at 1, and all three of them are freely estimated:
  scenario (#8): none of η*_j_*_=1_, η*_j_*_=2_, or η*_j_*_=3_ is assumed at 1.

The settings of the eight fitting scenarios are presented in Table 1.

**Table 1:** The summary of transmission advantage estimates of different types of SARS-CoV-2 variants in Brazil under different scenarios

Scenario	Transmission advantage of				AIC	AICc	BIC	HQIC	Remarks
	Original variant	New emerging variant							
	484E/501N	484E/501Y	484K/501N	484K/501Y					
(#1)	1 (reference)	1 (assumed)	1 (assumed)	1 (assumed)	6895.6	6960.3	8915.2	7615.1	Baseline model
(#2)	1 (reference)	1 (assumed)	1 (assumed)	1.23 (1.04–1.41)	6888.7	6953.8	8914.4	7610.4	Two types of new variant are assumed to have no effect
(#3)	1 (reference)	1 (assumed)	1.05 (0.93–1.17)	1 (assumed)	6893.5	6958.6	8919.3	7615.2	
(#4)	1 (reference)	1.08 (0.78–1.41)	1 (assumed)	1 (assumed)	6893.5	6958.6	8919.3	7615.2	
(#5)	1 (reference)	1 (assumed)	1.12 (1.01–1.22)	1.28 (1.08–1.47)	6888.3	6953.8	8920.3	7612.2	One type of new variant is assumed to have no effect
(#6)	1 (reference)	1.15 (0.83–1.52)	1 (assumed)	1.21 (1.05–1.42)	6889.9	6955.4	8921.9	7613.8	
(#7)	1 (reference)	1.08 (0.79–1.43)	1.04 (0.92–1.16)	1 (assumed)	6895.3	6960.8	8927.2	7619.2	
(#8)	1 (reference)	1.26 (0.92–1.66)	1.14 (1.03–1.27)	1.33 (1.13–1.56)	6888.4	6954.3	8926.5	7614.5	Full model

AIC = Akaike information criterion; AICc = corrected AIC for small sample size; BIC = Bayesian information criterion; HQIC = Hannan-Quinn information criterion. The highlighted scenario (#2) is selected as the main result, and shown in Figure 1.

We conduct the model fitting and parameter estimation under each scenario. The scenario with the best fitting performance is selected according to lowest values of 4 different information criteria including Akaike information criterion (AIC), corrected AIC for small sample size (AICc), Bayesian information criterion (BIC), and Hannan-Quinn information criterion (HQIC).

### Sensitivity analysis.

Sensitivity analysis was conducted to examine the robustness and significance of the determine transmission advantage estimates, that is, ηs. We examine the consistency of both directions of the effects and their 95% confidence intervals (CI) under alternative settings. The following three sensitivity checking schemes are performed.

For the first scheme, we consider a univariate logistic regression model between the overall *R_t_* as response and ρ*_j_*_=1,_*_t_*, ρ*_j_*_=2,_*_t_*, and ρ*_j_*_=3,_*_t_* as regressors. The regression coefficients of all ρ*_j_*_,_*_t_* are evaluated as the effect size of SARS-CoV2 variants on the overall transmissibility of COVID-19. For the second scheme, we repeat the estimating process of transmission advantage with alternative PDF of GT, that is, *w*(·), which is introduced in the previous section, “Variant-specific reproduction number.” We consider shorter and longer versions of mean GT at 4 days,^53^^,^^68^ and 7.5 days,^2^ respectively. For the third scheme, we repeat the analysis by replacing the Poisson-distributed likelihood function in Eq. (4) with a negative binomial (NB) distribution to further account for the superspreading potential of COVID-19 transmission. For the setting of NB distribution, we fixed the dispersion parameter at 0.4, which follows the estimation in recent studies.^55^^,^^69^^–^^74^

## RESULTS AND DISCUSSION

As of this writing, in Brazil, the epidemic curve had grown since March 2020 with two major epidemic waves, the first in August 2020, and the second is ongoing (Figure 1A). The original 484E/501N variants started being replaced by the other three types of variants in October 2020 and had almost vanished in Brazil after January 2021 (Figure 1C). In January 2021, the prevalence of the emerging 484K variants was 71.1% and 46.6% for 501Y variants. The prevalence of 484E/501Y (*j* = 1 type) variants is 10.1%, 34.6% for 484K/501N (*j* = 2 type) variants, and 36.5% for 484K/501Y (*j* = 3 type) variants (Figure 1D–F). As such, the linkage disequilibrium (LD) is calculated at 0.03, which indicates the occurrence of two mutations is likely random. Specially, the 484K/501Y variants are classified into the B.1.1.28.1 (or P.1, or 20J/501Y.V3) lineage.

For the eight fitting scenarios summarized in Table 1, we find that the transmission advantage of 484K/501Y (η*_j_*_=3_) variants is estimated larger than 1 significantly and consistently. In contrast, the scale of η*_j_*_=1_ for 484E/501Y variants appears statistically unclear compared with 1, that is, not significantly larger than 1. For the model selection, scenario 2 has the lowest AICc, BIC, and HQIC, and scenario 5 has the lowest AIC, and AICc. Both scenarios 2 and 5 also have close values of AIC with a difference of only 0.4. As such, scenario 2 is considered the main result and thus is presented in Figure 1B.

The modeling framework in this study links the mutation activity at molecular scale and COVID-19 transmissibility at population scale. We reconstruct the instantaneous reproduction numbers (*R_t_*) of COVID-19 cases infected by 484K/501Y variants and other variants in Brazil under fitting scenario 2 (Figure 1B). The overall trends of reproduction numbers are relatively high in the early phase of outbreak before and in May 2020 and the November 2020 for the second major epidemic wave, but gradually decrease thereafter. The average scale of reproduction number during the early outbreak is largely consistent with previous estimates.^2^^,^^3^^,^^75^^–^^77^

We report the estimated proportions of four types of SARS-CoV-2 variants, **E**[ρ*_t_*], fit the observed sequencing data well (Figure 1C–F). We infer the transmission advantage η*_j_*_=3_ for 484K/501Y variants at 1.23 (95% CI: 1.04–1.41), which means the E484K and N501Y substitutions together increase 23% of COVID-19’s transmissibility in Brazil, whereas other emerging variants, 484E/501Y and 484K/501N, are unlikely to have significant transmission advantage. Thus, in Figure 1B, the reproduction number of the 484K/501Y variant appears higher than that of the other genotypes (non-484K/501Y, or non-Gamma variants). For sensitivity checking, we find that the η*_j_*_=3_ estimates are consistently and significantly larger than 1 in similar scales as the main estimates (data not shown), which validates our findings.

We focus on the second major epidemic wave because E484K and N501Y substitutions emerged during the same period. Although the reproduction number of non-484K/501Y variants has fluctuated around 1 since December 2020, the reproduction number, *R_j_*_=3_, of 484K/501Y variants are largely greater than 1 during the same period, which has led to a large epidemic wave in Brazil in 2021 (see Figure 1A). Given that 484K/501Y variants trend to reach fixation, both the herd immunity threshold and intrinsic growth rate of epidemic may increase, which was previously discussed regarding the situation in United Kingdom,^12^^,^^25^ and thus the local nonpharmaceutical interventions for COVID-19 control may be enforced. Hence, we highlight the importance of our analytical framework, such that the public health risks related to viral mutations may be controllable with early preparedness.

The increase in transmissibility associated with the 484K/501Y variants is biologically reasonable. The N501Y substitution is a mutation on a key contact residue in the receptor binding domain on the S protein^78^ and is found to increase the ability of human angiotensin-converting enzyme 2 binding and cell infectivity in animal models,^79^ which appears similar to the previous D614G substitution.^80^ The E484K substitution is also located in the viral RBD and confers resistance to several monoclonal antibodies by affecting the binding process.^6^^,^^81^^,^^82^ However, according to the η*_j_*_=1_ estimates in Table 1, N501Y substitution alone appears less likely to form an advantage in fitness without accompanying 484K. Similarly, E484K substitution alone also might not secure a concrete transmission advantage robustly or significantly. Our finding indicates that the combined impact of 484K/501Y outperforms their independent effects, which implies statistically an interactive relationship.

Previous studies reported a higher case fatality risk among the individuals infected by SARS-CoV-2 strains in the B.1.1.7 lineage,^42^^,^^83^ some of which carry both E484K and N501Y substitutions. Given the transmission advantage of 484K/501Y (i.e., η*_j_*_=3_ > 1), the increasing intensity of COVID-19–related mortality is a public health concern. Clinical severity remains largely unassessed for the B.1.1.28 lineage in Brazil, and unexpected clinical outcomes may warrant adjustments in the treatment strategies. The emergence of 484K/501Y variants and its mutations (e.g., K417N or V1176F), combined with other VoC in Brazil and other places, implies the capacity of SARS-CoV-2 to evolve new phenotypes rapidly.^84^ Although the neutralizing level of BNT162b2 vaccine-elicited sera is recently found to be satisfactory for 484K/501Y variants,^20^ further investigation is required for other vaccine candidates at population scale.

This study has the following limitations. First, our analysis was based on the sequence data released in GISAID and thus is subject to the selection bias of sequences being released to the public domine.^12^ Second, the reconstruction of reproduction numbers relies on the setting of the generation time (GT). Theoretically, the GT distribution might be altered by the mutated strains. However, by screening the literature, we find no evidence that GT is associated with the E484K or N501Y substitution in SARS-CoV-2, and thus we model GT distribution of COVID-19 *w*(·) as a fixed Gamma distribution, following previous studies.^50^^–^^54^ Third, we consider *w*(·) as a fixed distribution. In the real-world situation, the time interval between transmission generations might vary,^75^^,^^85^ which may affect the reconstruction of the reproduction number. However, the long-term trends of *R_t_* estimates are unlikely to change due to slight variation in GT.^75^^,^^86^ Thus, we consider the impact of this limitation on the inference of transmission advantage may be negligible, and our model can be extended to a more complex context with the time-varying GT data available. Fourth, due to the lack of data from different Brazilian regions, we aggregated the national COVID-19 cases in Brazil to reconstruct the reproduction number series (Figure 1B). We acknowledge this analytical scheme neglects the heterogeneities in epidemiological characteristics of COVID-19 transmission,^55^^,^^74^^,^^87^^,^^88^ geographic separation in SARS-CoV-2 variants,^89^ individual response and vulnerability to COVID-19,^90^^–^^92^ and various nonpharmaceutical interventions^93^^,^^94^ for wide regions across different Brazilian locations. We note that the transmission advantage may vary under different local settings or situations. Fifth, ideally, *C*(*t*) in the *R_t_* estimation should be the number of COVID-19 cases with onset at time *t*. However, because surveillance data by date of onset are unavailable, we adapted the current dataset by reporting data as a proxy for the COVID-19 incidence time series. If one considers a constant reporting lag, the *R_t_* estimates will have the same trends but are shifted for this lag. Considering that a similar reporting delay also occurred during the collection of SARS-CoV-2 sequencing data, the effects of the two reporting lags could be counteracted. We believe that this approximation is unlikely to affect the main conclusions of this study. Furthermore, with detailed reporting lag of information for each individual case, adjustments for reporting delay can be carried out based on our current analytical framework. Sixth, this study focuses on exploring the effects on changing disease transmissibility associated with mutation activities, but real-world biological mechanisms, which are usually more complex, remain uncovered. Future studies are needed to explore the mechanisms of how E484K and N501Y mutations in SARS-CoV-2 affect the transmissibility of COVID-19. Seventh, there exist other mutations in the B.1.1.28 lineage, such as K417N and V1176F, but we merely considered the E484K and N501Y because they are dominant in B.1.1.28.1 and B.1.1.28.2 lineages, respectively. Given the lack of individual patient information, time-series data were used in this work, which means there is information loss from the data aggregation. As pointed out in previous studies,^83^ the independent effects of each commutation may not be disentangled in this study due to identification issues that the samples might fail to inform each estimate. Eighth, the interpretation of our findings should be limited to the COVID-19 epidemics in Brazil, but similar investigations could be conducted for other regions. Ninth, for simplification, we consider the transmission advantage (η) of new variants versus the wildtype as constant over time. This model assumption may not necessarily be strictly held considering several real-world determinants, including the accumulation of population immunity against different strains,^24^ selection pressure due to intervention strategies,^27^^,^^58^ and behavioral factors related to disease spread and transmission.^95^ Alternatively, the estimating framework of η can be extended into a real-time basis. Lastly, as a data-driven study, the estimated association should be treated with caution. In an ecological setting,^13^ although our analysis provides statistical evidence on the likelihood of causality, the findings in this study cannot guarantee causality, which requires further biomedical experiments for verification.

## Supplemental Material


Supplemental materials

## References

[1] Hu B Guo H Zhou P Shi ZL 2021. Characteristics of SARS-CoV-2 and COVID-19. Nat Rev Microbiol 19: 141–154. 33024307 10.1038/s41579-020-00459-7 PMC7537588

[2] Li Q 2020. Early transmission dynamics in Wuhan, China, of novel coronavirus-infected pneumonia. N Engl J Med 382: 1199–1207. 31995857 10.1056/NEJMoa2001316 PMC7121484

[3] Wu JT Leung K Leung GM 2020. Nowcasting and forecasting the potential domestic and international spread of the 2019-nCoV outbreak originating in Wuhan, China: a modelling study. Lancet 395: 689–697. 32014114 10.1016/S0140-6736(20)30260-9 PMC7159271

[4] Kutter JS Spronken MI Fraaij PL Fouchier RA Herfst S 2018. Transmission routes of respiratory viruses among humans. Curr Opin Virol 28: 142–151. 29452994 10.1016/j.coviro.2018.01.001 PMC7102683

[5] Fraser C Riley S Anderson RM Ferguson NM 2004. Factors that make an infectious disease outbreak controllable. Proc Natl Acad Sci USA 101: 6146–6151. 15071187 10.1073/pnas.0307506101 PMC395937

[6] Baum A 2020. Antibody cocktail to SARS-CoV-2 spike protein prevents rapid mutational escape seen with individual antibodies. Science 369: 1014–1018. 32540904 10.1126/science.abd0831 PMC7299283

[7] Tsetsarkin KA Vanlandingham DL McGee CE Higgs S 2007. A single mutation in chikungunya virus affects vector specificity and epidemic potential. PLoS Pathog 3: e201. 18069894 10.1371/journal.ppat.0030201 PMC2134949

[8] Tang JW Tambyah PA Hui DS 2021. Emergence of a new SARS-CoV-2 variant in the UK. J Infect 82: e27–e28. 10.1016/j.jinf.2020.12.024 PMC7834693 33383088

[9] Tang JW Toovey OTR Harvey KN Hui DDS 2021. Introduction of the South African SARS-CoV-2 variant 501Y. V2 into the UK. J Infect 82: e8–e10. 10.1016/j.jinf.2021.01.007 PMC7813514 33472093

[10] Claro IM 2021. Local Transmission of SARS-CoV-2 lineage B.1.1.7, Brazil, December 2020. Emerg Infect Dis 27: 970–972. 33496249 10.3201/eid2703.210038 PMC7920684

[11] Galloway SE 2021. Emergence of SARS-CoV-2 b. 1.1. 7 lineage—United States, December 29, 2020–January 12, 2021. Morb Mortal Wkly Rep 70: 95. 10.15585/mmwr.mm7003e2 PMC7821772 33476315

[12] Leung K Shum MH Leung GM Lam TT Wu JT 2021. Early transmissibility assessment of the N501Y mutant strains of SARS-CoV-2 in the United Kingdom, October to November 2020. Euro Surveill 26: 2002106. 10.2807/1560-7917.ES.2020.26.1.2002106 PMC7791602 33413740

[13] Graham MS et al., 2021. Changes in symptomatology, reinfection, and transmissibility associated with the SARS-CoV-2 variant B.1.1.7: an ecological study. *The Lancet Public Health* *6:* e335–e345. 10.1016/S2468-2667(21)00055-4 PMC8041365 33857453

[14] Public Health England , 2021. *Variants: Distribution of Cases Data: Variants of Concern or Under Investigation: Data up to 7 April 2021*. Available at: https://www.gov.uk/government/publications/covid-19-variants-genomically-confirmed-case-numbers/variants-distribution-of-cases-data.

[15] Toovey OTR Harvey KN Bird PW Tang JW-TW-T 2021. Introduction of Brazilian SARS-CoV-2 484K. V2 related variants into the UK. J Infect 82: e23–e24. 10.1016/j.jinf.2021.01.025 PMC7857057 33548358

[16] Voloch CM 2021. Genomic characterization of a novel SARS-CoV-2 lineage from Rio de Janeiro, Brazil. J Virol 95: e00119–e21. 10.1128/JVI.00119-21 PMC8139668 33649194

[17] Wise J 2021. COVID-19: the E484K mutation and the risks it poses. BMJ 372: n359. 33547053 10.1136/bmj.n359

[18] Walensky RP Walke HT Fauci AS 2021. SARS-CoV-2 variants of concern in the United States—challenges and opportunities. JAMA 325: 1037–1038. 33595644 10.1001/jama.2021.2294 PMC9009864

[19] Rondinone V 2021. VOC 202012/01 variant is effectively neutralized by antibodies produced by patients infected before its diffusion in Italy. Viruses-Basel 13: 276. 10.3390/v13020276 PMC7916909 33670182

[20] Xie XP 2021. Neutralization of SARS-CoV-2 spike 69/70 deletion, E484K and N501Y variants by BNT162b2 vaccine-elicited sera. Nat Med 27: 620–621. 33558724 10.1038/s41591-021-01270-4

[21] Moore JP Offit PA 2021. SARS-CoV-2 vaccines and the growing threat of viral variants. JAMA 325: 821–822. 33507218 10.1001/jama.2021.1114

[22] Muik A 2021. Neutralization of SARS-CoV-2 lineage B. 1.1. 7 pseudovirus by BNT162b2 vaccine–elicited human sera. Science 371: 1152–1153. 33514629 10.1126/science.abg6105 PMC7971771

[23] Supasa P 2021. Reduced neutralization of SARS-CoV-2 B. 1.1. 7 variant by convalescent and vaccine sera. Cell 184: 2201–2211. 33743891 10.1016/j.cell.2021.02.033 PMC7891044

[24] Faria NR 2021. Genomics and epidemiology of the P. 1 SARS-CoV-2 lineage in Manaus, Brazil. Science 372: 815–821. 33853970 10.1126/science.abh2644 PMC8139423

[25] Zhao S 2021. Quantifying the transmission advantage associated with N501Y substitution of SARS-CoV-2 in the UK: an early data-driven analysis. J Travel Med 28: taab011. 33506254 10.1093/jtm/taab011 PMC7928809

[26] Davies NG 2021. Estimated transmissibility and impact of SARS-CoV-2 lineage B.1.1.7 in England. Science 372: eabg3055. 33658326 10.1126/science.abg3055 PMC8128288

[27] Volz E 2021. Assessing transmissibility of SARS-CoV-2 lineage B. 1.1. 7 in England. Nature 593: 266–269. 33767447 10.1038/s41586-021-03470-x

[28] Volz E 2021. Evaluating the effects of SARS-CoV-2 spike mutation D614G on transmissibility and pathogenicity. Cell 184: 64–75 e11. 33275900 10.1016/j.cell.2020.11.020 PMC7674007

[29] Hou YJ 2020. SARS-CoV-2 D614G variant exhibits efficient replication ex vivo and transmission in vivo. Science 370: 1464–1468. 33184236 10.1126/science.abe8499 PMC7775736

[30] Zhang L 2020. SARS-CoV-2 spike-protein D614G mutation increases virion spike density and infectivity. Nat Commun 11: 6013. 33243994 10.1038/s41467-020-19808-4 PMC7693302

[31] Zhao S 2021. Modelling the association between COVID-19 transmissibility and D614G substitution in SARS-CoV-2 spike protein: using the surveillance data in California as an example. Theor Biol Med Model 8: 10. 10.1186/s12976-021-00140-3 PMC7941367 33750399

[32] da Silva Francisco R Jr 2021. Pervasive transmission of E484K and emergence of VUI-NP13L with evidence of SARS-CoV-2 co-infection events by two different lineages in Rio Grande do Sul, Brazil. Virus Res 296: 198345. 33631222 10.1016/j.virusres.2021.198345 PMC7898980

[33] Khan A Zia T Suleman M Khan T Ali SS Abbasi AA Mohammad A Wei DQ 2021. Higher infectivity of the SARS‐CoV‐2 new variants is associated with K417N/T, E484K, and N501Y mutants: an insight from structural data. J Cell Physiol 236: 7045–7057. 33755190 10.1002/jcp.30367 PMC8251074

[34] Hie B Zhong ED Berger B Bryson B 2021. Learning the language of viral evolution and escape. Science 371: 284–288. 33446556 10.1126/science.abd7331

[35] Gog JR Rimmelzwaan GF Osterhaus ADME Grenfell BT 2003. Population dynamics of rapid fixation in cytotoxic T lymphocyte escape mutants of influenza A. Proc Natl Acad Sci USA 100: 11143–11147. 12954978 10.1073/pnas.1830296100 PMC196941

[36] Smith DJ Lapedes AS de Jong JC Bestebroer TM Rimmelzwaan GF Osterhaus AD Fouchier RA 2004. Mapping the antigenic and genetic evolution of influenza virus. Science 305: 371–376. 15218094 10.1126/science.1097211

[37] Zhao S Lou J Cao L Chen Z Chan RW Chong MK Zee BC Chan PK Wang MH 2020. Quantifying the importance of the key sites on haemagglutinin in determining the selection advantage of influenza virus: using A/H3N2 as an example. J Infect 81: 452–482. 10.1016/j.jinf.2020.05.066 32504744

[38] Berkhoff EGM Boon ACM Nieuwkoop NJ Fouchier RAM Sintnicolaas K Osterhaus A Rimmelzwaan GF 2004. A mutation in the HLA-B* 2705-restricted NP383-391 epitope affects the human influenza A virus-specific cytotoxic T-lymphocyte response in vitro. J Virol 78: 5216–5222. 15113903 10.1128/JVI.78.10.5216-5222.2004 PMC400375

[39] Rimmelzwaan GF Berkhoff EGM Nieuwkoop NJ Fouchier RAM Osterhaus A 2004. Functional compensation of a detrimental amino acid substitution in a cytotoxic-T-lymphocyte epitope of influenza a viruses by comutations. J Virol 78: 8946–8949. 15280506 10.1128/JVI.78.16.8946-8949.2004 PMC479054

[40] Rimmelzwaan GF Berkhoff EGM Nieuwkoop NJ Smith DJ Fouchier RAM Osterhaus A 2005. Full restoration of viral fitness by multiple compensatory co-mutations in the nucleoprotein of influenza A virus cytotoxic T-lymphocyte escape mutants. J Gen Virol 86: 1801–1805. 15914859 10.1099/vir.0.80867-0

[41] Ferguson NM Cummings DA Cauchemez S Fraser C Riley S Meeyai A Iamsirithaworn S Burke DS 2005. Strategies for containing an emerging influenza pandemic in Southeast Asia. Nature 437: 209–214. 16079797 10.1038/nature04017

[42] Davies NG 2021. Increased mortality in community-tested cases of SARS-CoV-2 lineage B.1.1.7. Nature *593:* 270–274. 10.1038/s41586-021-03426-1 PMC9170116 33723411

[43] Shu Y McCauley J 2017. GISAID: global initiative on sharing all influenza data—from vision to reality. Euro Surveill 22: 30494. 28382917 10.2807/1560-7917.ES.2017.22.13.30494 PMC5388101

[44] Katoh K Rozewicki J Yamada KD 2019. MAFFT online service: multiple sequence alignment, interactive sequence choice and visualization. Brief Bioinform 20: 1160–1166. 28968734 10.1093/bib/bbx108 PMC6781576

[45] World Health Organization , 2021. Coronavirus Disease 2019 (COVID-19) Situation Reports. Geneva, Switzerland: World Health Organization. Available at: https://www.who.int/emergencies/diseases/novel-coronavirus-2019/situation-reports.

[46] Cori A Ferguson NM Fraser C Cauchemez S 2013. A new framework and software to estimate time-varying reproduction numbers during epidemics. Am J Epidemiol 178: 1505–1512. 24043437 10.1093/aje/kwt133 PMC3816335

[47] Zhao S Musa SS Hebert JT Cao P Ran J Meng J He D Qin J 2020. Modelling the effective reproduction number of vector-borne diseases: the yellow fever outbreak in Luanda, Angola 2015–2016 as an example. PeerJ 8: e8601. 32149023 10.7717/peerj.8601 PMC7049463

[48] Champredon D Dushoff J Earn DJD 2018. Equivalence of the Erlang-distributed SEIR epidemic model and the renewal equation. SIAM J Appl Math 78: 3258–3278.

[49] Wallinga J Lipsitch M 2007. How generation intervals shape the relationship between growth rates and reproductive numbers. Proc Biol Sci 274: 599–604. 17476782 10.1098/rspb.2006.3754 PMC1766383

[50] Ferretti L Wymant C Kendall M Zhao L Nurtay A Abeler-Dorner L Parker M Bonsall D Fraser C 2020. Quantifying SARS-CoV-2 transmission suggests epidemic control with digital contact tracing. Science 368: eabb6936. 32234805 10.1126/science.abb6936 PMC7164555

[51] Ganyani T Kremer C Chen D Torneri A Faes C Wallinga J Hens N 2020. Estimating the generation interval for coronavirus disease (COVID-19) based on symptom onset data, March 2020. Euro Surveill 25: 2000257. 10.2807/1560-7917.ES.2020.25.17.2000257 PMC7201952 32372755

[52] Tindale LC 2020. Evidence for transmission of COVID-19 prior to symptom onset. eLife 9: e57149. 32568070 10.7554/eLife.57149 PMC7386904

[53] Zhao S 2020. Estimating the time interval between transmission generations when negative values occur in the serial interval data: using COVID-19 as an example. Math Biosci Eng 17: 3512–3519. 32987541 10.3934/mbe.2020198

[54] He X 2020. Temporal dynamics in viral shedding and transmissibility of COVID-19. Nat Med 26: 672–675. 32296168 10.1038/s41591-020-0869-5

[55] Adam DC Wu P Wong JY Lau EHY Tsang TK Cauchemez S Leung GM Cowling BJ 2020. Clustering and superspreading potential of SARS-CoV-2 infections in Hong Kong. Nat Med 26: 1714–1719. 32943787 10.1038/s41591-020-1092-0

[56] Biggerstaff M 2020. Early Insights from statistical and mathematical modeling of key epidemiologic parameters of COVID-19. Emerg Infect Dis J 26. 10.3201/eid2611.201074 PMC7588530 32917290

[57] Zhao S 2021. Estimating the generation interval and inferring the latent period of COVID-19 from the contact tracing data. Epidemics 36: 100482. 34175549 10.1016/j.epidem.2021.100482 PMC8223005

[58] Leung K Lipsitch M Yuen KY Wu JT 2017. Monitoring the fitness of antiviral-resistant influenza strains during an epidemic: a mathematical modelling study. Lancet Infect Dis 17: 339–347. 27914853 10.1016/S1473-3099(16)30465-0 PMC5470942

[59] Fan JQ Huang T 2005. Profile likelihood inferences on semiparametric varying-coefficient partially linear models. Bernoulli 11: 1031–1057.

[60] Bolker BM 2008. Ecological Models and Data in R. Princeton, NJ: Princeton University Press.

[61] Breto C He DH Ionides EL King AA 2009. Time series analysis via mechanistic models. Ann Appl Stat 3: 319–348.

[62] He D Ionides EL King AA 2010. Plug-and-play inference for disease dynamics: measles in large and small populations as a case study. J R Soc Interface 7: 271–283. 19535416 10.1098/rsif.2009.0151 PMC2842609

[63] Lin Q Chiu AP Zhao S He D 2018. Modeling the spread of Middle East respiratory syndrome coronavirus in Saudi Arabia. Stat Methods Med Res 27: 1968–1978. 29846148 10.1177/0962280217746442

[64] Zhao S 2020. Estimating the serial interval of the novel coronavirus disease (COVID-19): a statistical analysis using the public data in Hong Kong from January 16 to February 15, 2020. Front Phys 8: 347.

[65] Zhao S Lou Y Chiu APY He D 2018. Modelling the skip-and-resurgence of Japanese encephalitis epidemics in Hong Kong. J Theor Biol 454: 1–10. 29792875 10.1016/j.jtbi.2018.05.017 PMC7094098

[66] Wang K 2020. Estimating the serial interval of the novel coronavirus disease (COVID-19) based on the public surveillance data in Shenzhen, China, from 19 January to 22 February 2020. Transbound Emerg Dis 67: 2818–2822. 32452648 10.1111/tbed.13647 PMC7283843

[67] Zhao S 2021. An early assessment of a case fatality risk associated with P.1 SARS-CoV-2 lineage in Brazil: an ecological study. J Travel Med (In press). doi: 10.1093/jtm/taab078. PMC8344495 34155521

[68] Du Z Xu X Wu Y Wang L Cowling BJ Meyers LA 2020. Serial interval of COVID-19 among publicly reported confirmed cases. Emerg Infect Dis 26: 1341–1343. 32191173 10.3201/eid2606.200357 PMC7258488

[69] Bi Q 2020. Epidemiology and transmission of COVID-19 in 391 cases and 1286 of their close contacts in Shenzhen, China: a retrospective cohort study. Lancet Infect Dis 20: 911–919. 32353347 10.1016/S1473-3099(20)30287-5 PMC7185944

[70] Endo A Abbott S Kucharski AJ Funk S 2020. Estimating the overdispersion in COVID-19 transmission using outbreak sizes outside China. Wellcome Open Res 5: 67. 32685698 10.12688/wellcomeopenres.15842.1 PMC7338915

[71] Lau MSY Grenfell B Thomas M Bryan M Nelson K Lopman B 2020. Characterizing superspreading events and age-specific infectiousness of SARS-CoV-2 transmission in Georgia, USA. Proc Natl Acad Sci USA 117: 22430–22435. 32820074 10.1073/pnas.2011802117 PMC7486752

[72] Tariq A Lee Y Roosa K Blumberg S Yan P Ma S Chowell G 2020. Real-time monitoring the transmission potential of COVID-19 in Singapore, March 2020. BMC Med 18: 166. 32493466 10.1186/s12916-020-01615-9 PMC7268586

[73] Zhang Y Li Y Wang L Li M Zhou X 2020. Evaluating transmission heterogeneity and super-spreading event of COVID-19 in a metropolis of China. Int J Environ Res Public Health 17: 3705. 10.3390/ijerph17103705 PMC7277812 32456346

[74] Zhao S 2021. Inferencing superspreading potential using zero-truncated negative binomial model: exemplification with COVID-19. BMC Med Res Methodol 21: 30. 33568100 10.1186/s12874-021-01225-w PMC7874987

[75] Ali ST Wang L Lau EHY Xu XK Du Z Wu Y Leung GM Cowling BJ 2020. Serial interval of SARS-CoV-2 was shortened over time by nonpharmaceutical interventions. Science 369: 1106–1109. 32694200 10.1126/science.abc9004 PMC7402628

[76] Chinazzi M 2020. The effect of travel restrictions on the spread of the 2019 novel coronavirus (COVID-19) outbreak. Science 368: 395–400. 32144116 10.1126/science.aba9757 PMC7164386

[77] Gatto M Bertuzzo E Mari L Miccoli S Carraro L Casagrandi R Rinaldo A 2020. Spread and dynamics of the COVID-19 epidemic in Italy: effects of emergency containment measures. Proc Natl Acad Sci USA 117: 10484–10491. 32327608 10.1073/pnas.2004978117 PMC7229754

[78] Starr TN 2020. Deep mutational scanning of SARS-CoV-2 receptor binding domain reveals constraints on folding and ACE2 binding. Cell 182: 1295–1310.e20. 32841599 10.1016/j.cell.2020.08.012 PMC7418704

[79] Gu H 2020. Adaptation of SARS-CoV-2 in BALB/c mice for testing vaccine efficacy. Science 369: 1603–1607. 32732280 10.1126/science.abc4730 PMC7574913

[80] Yurkovetskiy L 2020. Structural and functional analysis of the D614G SARS-CoV-2 spike protein variant. Cell 183: 739–751 e8. 32991842 10.1016/j.cell.2020.09.032 PMC7492024

[81] Ku Z 2021. Molecular determinants and mechanism for antibody cocktail preventing SARS-CoV-2 escape. Nat Commun 12: 1–13. 33473140 10.1038/s41467-020-20789-7 PMC7817669

[82] Wang Q 2020. Structural and functional basis of SARS-CoV-2 entry by using human ACE2. Cell 181: 894–904. 32275855 10.1016/j.cell.2020.03.045 PMC7144619

[83] Zhao S 2021. Inferring the association between the risk of COVID-19 case fatality and N501Y substitution in SARS-CoV-2. Viruses 13: 638. 33918060 10.3390/v13040638 PMC8070306

[84] Waters E Doyle J 2004. Systematic reviews of public health in developing countries are in train. BMJ 328: 585. 10.1136/bmj.328.7439.585 PMC381063 15001523

[85] Zhao S 2020. COVID-19 and gender-specific difference: analysis of public surveillance data in Hong Kong and Shenzhen, China, from January 10 to February 15, 2020. Infect Control Hosp Epidemiol 41: 750–751. 32146921 10.1017/ice.2020.64 PMC7113032

[86] Torneri A Libin P Scalia Tomba G Faes C Wood JG Hens N 2021. On realized serial and generation intervals given control measures: the COVID-19 pandemic case. PLOS Comput Biol 17: e1008892. 33780436 10.1371/journal.pcbi.1008892 PMC8031880

[87] Sun K 2021. Transmission heterogeneities, kinetics, and controllability of SARS-CoV-2. Science 371: eabe2424. 33234698 10.1126/science.abe2424 PMC7857413

[88] Leung K Wu JT Liu D Leung GM 2020. First-wave COVID-19 transmissibility and severity in China outside Hubei after control measures, and second-wave scenario planning: a modelling impact assessment. Lancet 395: 1382–1393. 32277878 10.1016/S0140-6736(20)30746-7 PMC7195331

[89] Ong SWX Young BE Lye DC 2021. Lack of detail in population-level data impedes analysis of SARS-CoV-2 variants of concern and clinical outcomes. Lancet Infect Dis 21: 1195–1197. 33857407 10.1016/S1473-3099(21)00201-2 PMC8041357

[90] Zhao S Stone L Gao D Musa SS Chong MKC He D Wang MH 2020. Imitation dynamics in the mitigation of the novel coronavirus disease (COVID-19) outbreak in Wuhan, China from 2019 to 2020. Ann Transl Med 8: 448. 32395492 10.21037/atm.2020.03.168 PMC7210122

[91] Sun K Chen J Viboud C 2020. Early epidemiological analysis of the coronavirus disease 2019 outbreak based on crowdsourced data: a population-level observational study. Lancet Digit Health 2: e201–e208. 32309796 10.1016/S2589-7500(20)30026-1 PMC7158945

[92] Jentsch PC Anand M Bauch CT 2021. Prioritising COVID-19 vaccination in changing social and epidemiological landscapes: a mathematical modelling study. Lancet Infect Dis (In press). 10.1016/S1473-3099(21)00057-8 PMC8012029 33811817

[93] Liu Y Morgenstern C Kelly J Lowe R Jit M 2021. The impact of non-pharmaceutical interventions on SARS-CoV-2 transmission across 130 countries and territories. BMC Med 19: 1–12. 33541353 10.1186/s12916-020-01872-8 PMC7861967

[94] Li Y Campbell H Kulkarni D Harpur A Nundy M Wang X Nair H, for Covid UN 2021. The temporal association of introducing and lifting non-pharmaceutical interventions with the time-varying reproduction number (R) of SARS-CoV-2: a modelling study across 131 countries. Lancet Infect Dis 21: 193–202. 33729915 10.1016/S1473-3099(20)30785-4 PMC7581351

[95] Kraemer MUG 2021. Spatiotemporal invasion dynamics of SARS-CoV-2 lineage B.1.1.7 emergence. Science 373: 889–895. 34301854 10.1126/science.abj0113 PMC9269003

